# 5-Propyl-6-(*p*-tolyl­sulfan­yl)pyrimidine-2,4(1*H*,3*H*)-dione

**DOI:** 10.1107/S1600536814000749

**Published:** 2014-01-22

**Authors:** Fatmah A. M. Al-Omary, Hazem A. Ghabbour, Ali A. El-Emam, C. S. Chidan Kumar, Hoong-Kun Fun

**Affiliations:** a Department of Pharmaceutical Chemistry, College of Pharmacy, King Saud University, PO Box 2457, Riaydh 11451, Saudi Arabia; b X-ray Crystallography Unit, School of Physics, Universiti Sains Malaysia, 11800 USM, Penang, Malaysia

## Abstract

In the title pymiridine-2,4-dione derivative, C_14_H_16_N_2_O_2_S, the dihedral angle between the six-membered rings is 66.69 (10)°. The mol­ecule is twisted about the C_p_—S (p = pyrimidine) bond, with a C—S—C—N torsion angle of −19.57 (16)°. In the crystal, adjacent mol­ecules form inversion dimers through pairs of strong N—H⋯O hydrogen bonds, generating an *R*_2_^2^(8) ring motif. The dimers are connected into chains extending along the *c-*axis direction through additional N—H⋯O hydrogen bonds.

## Related literature   

For the pharmacological activity of pyrimidine-2,4-dione derivatives, see: Al-Abdullah *et al.* (2011 ▶); El-Emam *et al.* (2004 ▶); Hopkins *et al.* (1996 ▶); Klein *et al.* (2001 ▶); Miyasaka *et al.* (1989 ▶); Nencka *et al.* (2006 ▶); Russ *et al.* (2003 ▶); Tanaka *et al.* (1995 ▶); For related pyrimidine-2,4-dione structures, see: El-Brollosy *et al.* (2009 ▶); Wang *et al.* (2006 ▶). For hydrogen-bond motifs, see: Bernstein *et al.* (1995 ▶). For reference bond lengths, see: Allen *et al.* (1987 ▶).
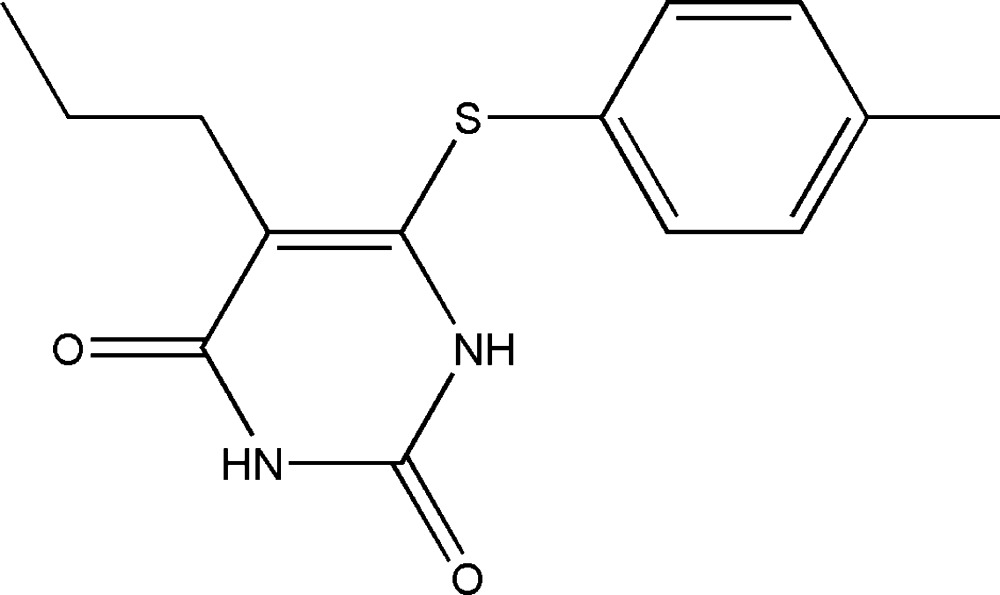


## Experimental   

### 

#### Crystal data  

C_14_H_16_N_2_O_2_S
*M* *_r_* = 276.35
Monoclinic, 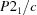

*a* = 11.8356 (3) Å
*b* = 10.3040 (2) Å
*c* = 13.3999 (3) Å
β = 119.850 (2)°
*V* = 1417.37 (6) Å^3^
*Z* = 4
Cu *K*α radiation
μ = 2.03 mm^−1^
*T* = 293 K
0.82 × 0.71 × 0.08 mm

#### Data collection  

Bruker APEXII CCD diffractometer
Absorption correction: multi-scan (*SADABS*; Bruker, 2009 ▶) *T*_min_ = 0.287, *T*_max_ = 0.855
9643 measured reflections
2658 independent reflections
2450 reflections with *I* > 2σ(*I*)
*R*_int_ = 0.024

#### Refinement  

*R*[*F* ^2^ > 2σ(*F* ^2^)] = 0.044
*wR*(*F* ^2^) = 0.126
*S* = 1.06
2658 reflections
180 parameters
H atoms treated by a mixture of independent and constrained refinement
Δρ_max_ = 0.33 e Å^−3^
Δρ_min_ = −0.39 e Å^−3^

### 

Data collection: *APEX2* (Bruker, 2009 ▶); cell refinement: *SAINT* (Bruker, 2009 ▶); data reduction: *SAINT*; program(s) used to solve structure: *SHELXTL* (Sheldrick, 2008 ▶); program(s) used to refine structure: *SHELXTL*; molecular graphics: *SHELXTL*; software used to prepare material for publication: *SHELXTL* and *PLATON* (Spek, 2009 ▶).

## Supplementary Material

Crystal structure: contains datablock(s) global, I. DOI: 10.1107/S1600536814000749/sj5382sup1.cif

Structure factors: contains datablock(s) I. DOI: 10.1107/S1600536814000749/sj5382Isup2.hkl

Supporting information file. DOI: 10.1107/S1600536814000749/sj5382Isup3.cml

CCDC reference:

Additional supporting information:  crystallographic information; 3D view; checkCIF report

## Figures and Tables

**Table 1:** Hydrogen-bond geometry (Å, °)

*D*—H⋯*A*	*D*—H	H⋯*A*	*D*⋯*A*	*D*—H⋯*A*
N2—H2*N*2⋯O1^i^	0.84 (2)	1.98 (2)	2.815 (2)	173 (2)
N1—H1*N*1⋯O2^ii^	0.79 (2)	2.17 (2)	2.8988 (18)	155 (2)

Symmetry codes: (i) 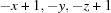; (ii) 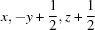.

**Fig. 1.**
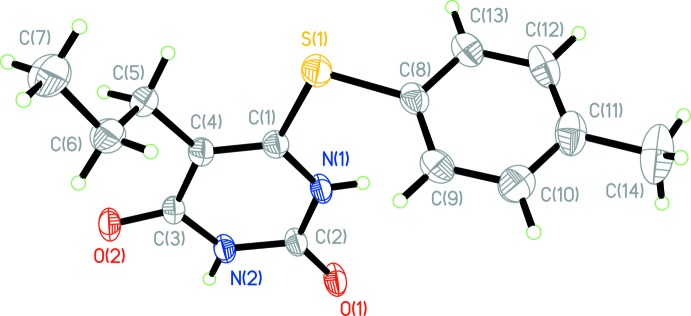
The molecular structure of the title compound with the atom labelling scheme and 30% probability displacement ellipsoids.

**Fig. 2.**
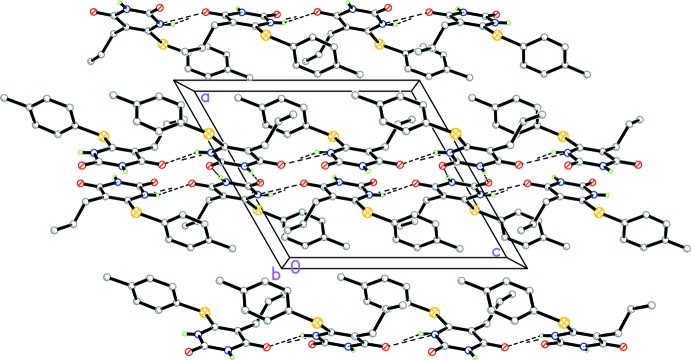
Crystal packing of the title compound, viewed along the b axis, showing the N1–H1N1···O2 and N2–H2N2···O1 intermolecular hydrogen bonds as dashed lines. H-atoms not involved in the hydrogen bonding are omited for clarity.

## supplementary crystallographic information

### 1. Comment

Pyrimidine-2,4-diones and their related derivatives have long been known for their diverse chemotherapeutic activities including antiviral activity against the HIV (Miyasaka *et al.*, 1989; Tanaka *et al.*, 1995; Hopkins *et al.*, 1996; El-Emam *et al.*, 2004), and HSV viruses (Russ *et al.*, 2003). In addition, potent anticancer activity was observed for several pyrimidine-2,4-diones (Klein *et al.*, 2001; Nencka *et al.*, 2006). In continuation to our interest in the chemical and pharmacological properties of pyrimidine and uracil derivatives (Al-Abdullah *et al.*, 2011; El-Brollosy *et al.*, 2009), we have synthesized the title compound (I) as a potential chemotherapeutic agent.

The title compound (I) is a derivative of pymiridine-2,4-dione. The heterocycle contains the structural unit CON_2_H_2_CO, forming the dihedral angle of 66.69 (10)° with the adjacent benzene ring. The molecule is bent (Fig. 1) at the S atom with a C–S–C–N torsion angle of -19.57 (16)°. The bond lengths (Allen *et al.*, 1987) and angles in the title compound are within normal ranges and are comparable with those reported earlier (El-Brollosy *et al.*, 2009; Wang *et al.*, 2006). The crystal structure features for two types of intermolecular N–H···O hydrogen bonds (Table 1). Two adjacent molecules form inversion-related dimers through strong N2–H2A···O1 hydrogen bonds (symmetry code: -*x* + 1, -*y*, -*z* + 1), generating an *R*_2_^2^(8) ring motif (Bernstein *et al.*, 1995) (Fig. 2). These dimers are further connected into chains extending along *c* axis through additional N1–H1N1···O2 hydrogen bonds (symmetry code: *x*, -*y* +1/2, *z* + 1/2) (Fig. 2). Crystal stability is mainly consolidated by these hydrogen bonding interactions forming two-dimensional networks parallel to the *bc* plane.

### 2. Experimental

A mixture of 6-chloro-5-propyluracil (943 mg, 0.005 mol), *p*-thiocresol (621 mg, 0.005 mol) and potassium hydroxide (281 mg, 0.005 mol), in ethanol (10 ml), was heated under reflux for 3 h. The solvent was then distilled off *in vacuo* and the residue was washed with cold water, dried and crystallized from ethanol to yield 995 mg (72%) of the title compound (C_14_H_16_N_2_O_2_S) as colorless needle-like crystals. M·P.: 446–448 K.

^1^H NMR (DMSO-d_6_, 500.13 MHz): δ 0.84 (t, 3H, CH_2_CH_3_, J = 7.0 Hz), 1.38–1.40 (m, 2H, CH_2_CH_3_), 2.31 (s, 3H, Ar—CH_3_), 2.44 (t, 2H, CH_2_CH_2_CH_3_, J = 7.0 Hz), 7.22 (d, 2H, Ar—H, J = 7.0 Hz), 7.30 (d, 2H, Ar—H, J = 7.0 Hz), 10.74 (s, 1H, NH), 11.21 (s, 1H, NH). ^13^C NMR (DMSO-d_6_, 125.76 MHz): δ 13.73 (CH_2_CH_3_), 20.54 (CH_2_CH_3_), 22.05 (CH_2_CH_2_CH_3_), 28.11 (Ar—CH_3_), 116.60 (pyrimidine C-5), 128.10, 130.01, 130.18, 130.55 (Ar—C), 143.84 (pyrimidine C-6), 150.46 (C=O), 163.19 (C=O).

### 3. Refinement

The nitrogen-bound H-atoms were located in a difference Fourier map and were refined freely. Other H atoms were positioned geometrically (C=H 0.93–0.97 Å) and refined using a riding model with *U*_iso_(H) = 1.2 *U*_eq_(C) or 1.5 *U*_eq_(C) for methyl H atoms. A rotating group model was used for the methyl group.

### Crystal data

**Table:**

C_14_H_16_N_2_O_2_S	*F*(000) = 584
*M**_r_* = 276.35	*D*_x_ = 1.295 Mg m^−^^3^
Monoclinic, *P*2_1_/*c*	Cu *K*α radiation, λ = 1.54178 Å
Hall symbol: -P 2ybc	Cell parameters from 5622 reflections
*a* = 11.8356 (3) Å	θ = 3.8–69.5°
*b* = 10.3040 (2) Å	µ = 2.03 mm^−^^1^
*c* = 13.3999 (3) Å	*T* = 293 K
β = 119.850 (2)°	Needle, colourless
*V* = 1417.37 (6) Å^3^	0.82 × 0.71 × 0.08 mm
*Z* = 4	

### Data collection

**Table:**

Bruker APEXII CCD diffractometer	2658 independent reflections
Radiation source: fine-focus sealed tube	2450 reflections with *I* > 2σ(*I*)
Graphite monochromator	*R*_int_ = 0.024
φ and ω scans	θ_max_ = 69.9°, θ_min_ = 4.3°
Absorption correction: multi-scan (*SADABS*; Bruker, 2009)	*h* = −14→14
*T*_min_ = 0.287, *T*_max_ = 0.855	*k* = −12→10
9643 measured reflections	*l* = −16→16

### Refinement

**Table:**

Refinement on *F*^2^	Primary atom site location: structure-invariant direct methods
Least-squares matrix: full	Secondary atom site location: difference Fourier map
*R*[*F*^2^ > 2σ(*F*^2^)] = 0.044	Hydrogen site location: inferred from neighbouring sites
*wR*(*F*^2^) = 0.126	H atoms treated by a mixture of independent and constrained refinement
*S* = 1.06	*w* = 1/[σ^2^(*F*_o_^2^) + (0.0707*P*)^2^ + 0.439*P*] where *P* = (*F*_o_^2^ + 2*F*_c_^2^)/3
2658 reflections	(Δ/σ)_max_ < 0.001
180 parameters	Δρ_max_ = 0.33 e Å^−^^3^
0 restraints	Δρ_min_ = −0.39 e Å^−^^3^

### Special details

**Table:**

Geometry. All e.s.d.'s (except the e.s.d. in the dihedral angle between two l.s. planes) are estimated using the full covariance matrix. The cell e.s.d.'s are taken into account individually in the estimation of e.s.d.'s in distances, angles and torsion angles; correlations between e.s.d.'s in cell parameters are only used when they are defined by crystal symmetry. An approximate (isotropic) treatment of cell e.s.d.'s is used for estimating e.s.d.'s involving l.s. planes.
Refinement. Refinement of *F*^2^ against ALL reflections. The weighted *R*-factor *wR* and goodness of fit *S* are based on *F*^2^, conventional *R*-factors *R* are based on *F*, with *F* set to zero for negative *F*^2^. The threshold expression of *F*^2^ > σ(*F*^2^) is used only for calculating *R*-factors(gt) *etc*. and is not relevant to the choice of reflections for refinement. *R*-factors based on *F*^2^ are statistically about twice as large as those based on *F*, and *R*- factors based on ALL data will be even larger.

### Fractional atomic coordinates and isotropic or equivalent isotropic displacement parameters (Å^2^)

**Table:**

	*x*	*y*	*z*	*U*_iso_*/*U*_eq_	
S1	0.28497 (6)	0.53022 (5)	0.47950 (5)	0.0610 (2)	
O1	0.45217 (15)	0.07980 (13)	0.58320 (11)	0.0594 (4)	
N1	0.38171 (14)	0.28614 (14)	0.53086 (12)	0.0434 (3)	
C1	0.35088 (16)	0.38803 (16)	0.45499 (14)	0.0426 (4)	
O2	0.44741 (15)	0.24368 (14)	0.26889 (11)	0.0584 (4)	
N2	0.45477 (15)	0.16770 (15)	0.42914 (12)	0.0441 (3)	
C2	0.43133 (16)	0.17195 (16)	0.51850 (13)	0.0418 (4)	
C3	0.42669 (17)	0.26374 (17)	0.34806 (14)	0.0438 (4)	
C4	0.37290 (17)	0.38287 (17)	0.36542 (14)	0.0460 (4)	
C5	0.3446 (2)	0.49234 (19)	0.28137 (16)	0.0529 (4)	
H5A	0.4150	0.4980	0.2642	0.063*	
H5B	0.3424	0.5733	0.3173	0.063*	
C6	0.2176 (2)	0.4763 (2)	0.17021 (19)	0.0625 (5)	
H6A	0.1478	0.4628	0.1872	0.075*	
H6B	0.2225	0.4002	0.1300	0.075*	
C7	0.1865 (3)	0.5949 (3)	0.0927 (2)	0.0891 (8)	
H7A	0.1054	0.5816	0.0229	0.134*	
H7B	0.2547	0.6076	0.0746	0.134*	
H7C	0.1800	0.6702	0.1317	0.134*	
C8	0.22523 (18)	0.47959 (18)	0.57142 (16)	0.0489 (4)	
C9	0.1270 (2)	0.3881 (2)	0.53692 (18)	0.0586 (5)	
H9A	0.0937	0.3477	0.4657	0.070*	
C10	0.0791 (2)	0.3573 (2)	0.6090 (2)	0.0669 (6)	
H10A	0.0149	0.2941	0.5866	0.080*	
C11	0.1244 (2)	0.4185 (3)	0.7138 (2)	0.0694 (6)	
C12	0.2192 (2)	0.5123 (3)	0.74454 (19)	0.0720 (6)	
H12A	0.2485	0.5565	0.8136	0.086*	
C13	0.2718 (2)	0.5423 (2)	0.67553 (18)	0.0587 (5)	
H13A	0.3376	0.6040	0.6990	0.070*	
C14	0.0734 (3)	0.3817 (4)	0.7929 (3)	0.1124 (13)	
H14A	0.0086	0.3151	0.7575	0.169*	
H14B	0.0353	0.4564	0.8074	0.169*	
H14C	0.1438	0.3501	0.8643	0.169*	
H2N2	0.483 (2)	0.096 (2)	0.4206 (19)	0.055 (6)*	
H1N1	0.3791 (19)	0.291 (2)	0.5883 (19)	0.046 (5)*	

### Atomic displacement parameters (Å^2^)

**Table:**

	*U*^11^	*U*^22^	*U*^33^	*U*^12^	*U*^13^	*U*^23^
S1	0.0879 (4)	0.0444 (3)	0.0701 (4)	0.0183 (2)	0.0541 (3)	0.0103 (2)
O1	0.0977 (10)	0.0498 (7)	0.0494 (7)	0.0248 (7)	0.0507 (7)	0.0134 (6)
N1	0.0588 (8)	0.0447 (8)	0.0356 (7)	0.0101 (6)	0.0303 (6)	0.0027 (6)
C1	0.0483 (8)	0.0411 (9)	0.0399 (8)	0.0035 (7)	0.0231 (7)	0.0013 (7)
O2	0.0888 (9)	0.0579 (8)	0.0487 (7)	0.0058 (7)	0.0494 (7)	0.0040 (6)
N2	0.0609 (8)	0.0421 (8)	0.0392 (7)	0.0091 (6)	0.0323 (7)	0.0029 (6)
C2	0.0523 (8)	0.0434 (9)	0.0335 (7)	0.0081 (7)	0.0243 (7)	0.0018 (7)
C3	0.0545 (9)	0.0461 (9)	0.0367 (8)	−0.0002 (7)	0.0271 (7)	0.0009 (7)
C4	0.0560 (9)	0.0443 (9)	0.0414 (8)	0.0019 (7)	0.0270 (7)	0.0026 (7)
C5	0.0677 (11)	0.0465 (9)	0.0514 (10)	−0.0021 (8)	0.0348 (9)	0.0033 (8)
C6	0.0705 (12)	0.0537 (11)	0.0591 (12)	0.0046 (9)	0.0291 (10)	0.0076 (9)
C7	0.108 (2)	0.0711 (16)	0.0669 (14)	0.0175 (14)	0.0272 (14)	0.0261 (12)
C8	0.0557 (10)	0.0460 (10)	0.0498 (10)	0.0145 (7)	0.0299 (8)	0.0031 (7)
C9	0.0604 (11)	0.0551 (11)	0.0600 (11)	0.0097 (9)	0.0298 (9)	−0.0052 (9)
C10	0.0603 (11)	0.0616 (12)	0.0860 (15)	0.0110 (9)	0.0417 (11)	0.0096 (11)
C11	0.0625 (12)	0.0885 (16)	0.0680 (13)	0.0275 (12)	0.0407 (11)	0.0210 (12)
C12	0.0752 (14)	0.0937 (17)	0.0485 (11)	0.0216 (13)	0.0318 (10)	−0.0030 (11)
C13	0.0596 (11)	0.0623 (12)	0.0544 (11)	0.0090 (9)	0.0285 (9)	−0.0054 (9)
C14	0.099 (2)	0.167 (4)	0.102 (2)	0.038 (2)	0.0731 (19)	0.044 (2)

### Geometric parameters (Å, º)

**Table:**

S1—C1	1.7658 (17)	C6—H6B	0.9700
S1—C8	1.7766 (19)	C7—H7A	0.9600
O1—C2	1.225 (2)	C7—H7B	0.9600
N1—C2	1.361 (2)	C7—H7C	0.9600
N1—C1	1.378 (2)	C8—C13	1.379 (3)
N1—H1N1	0.79 (2)	C8—C9	1.385 (3)
C1—C4	1.350 (2)	C9—C10	1.378 (3)
O2—C3	1.220 (2)	C9—H9A	0.9300
N2—C2	1.358 (2)	C10—C11	1.381 (4)
N2—C3	1.382 (2)	C10—H10A	0.9300
N2—H2N2	0.84 (3)	C11—C12	1.378 (4)
C3—C4	1.454 (2)	C11—C14	1.508 (3)
C4—C5	1.509 (2)	C12—C13	1.383 (3)
C5—C6	1.509 (3)	C12—H12A	0.9300
C5—H5A	0.9700	C13—H13A	0.9300
C5—H5B	0.9700	C14—H14A	0.9600
C6—C7	1.526 (3)	C14—H14B	0.9600
C6—H6A	0.9700	C14—H14C	0.9600
			
C1—S1—C8	104.50 (8)	C6—C7—H7A	109.5
C2—N1—C1	122.71 (14)	C6—C7—H7B	109.5
C2—N1—H1N1	114.1 (16)	H7A—C7—H7B	109.5
C1—N1—H1N1	122.9 (16)	C6—C7—H7C	109.5
C4—C1—N1	121.90 (15)	H7A—C7—H7C	109.5
C4—C1—S1	119.78 (13)	H7B—C7—H7C	109.5
N1—C1—S1	118.30 (12)	C13—C8—C9	120.23 (19)
C2—N2—C3	126.53 (15)	C13—C8—S1	117.82 (16)
C2—N2—H2N2	114.9 (16)	C9—C8—S1	121.72 (15)
C3—N2—H2N2	118.3 (16)	C10—C9—C8	119.5 (2)
O1—C2—N2	122.78 (15)	C10—C9—H9A	120.3
O1—C2—N1	122.06 (14)	C8—C9—H9A	120.3
N2—C2—N1	115.16 (14)	C9—C10—C11	121.3 (2)
O2—C3—N2	119.25 (16)	C9—C10—H10A	119.3
O2—C3—C4	125.16 (16)	C11—C10—H10A	119.3
N2—C3—C4	115.59 (14)	C12—C11—C10	118.1 (2)
C1—C4—C3	117.98 (15)	C12—C11—C14	121.2 (3)
C1—C4—C5	124.38 (16)	C10—C11—C14	120.8 (3)
C3—C4—C5	117.64 (15)	C11—C12—C13	121.8 (2)
C4—C5—C6	113.39 (16)	C11—C12—H12A	119.1
C4—C5—H5A	108.9	C13—C12—H12A	119.1
C6—C5—H5A	108.9	C8—C13—C12	119.0 (2)
C4—C5—H5B	108.9	C8—C13—H13A	120.5
C6—C5—H5B	108.9	C12—C13—H13A	120.5
H5A—C5—H5B	107.7	C11—C14—H14A	109.5
C5—C6—C7	111.6 (2)	C11—C14—H14B	109.5
C5—C6—H6A	109.3	H14A—C14—H14B	109.5
C7—C6—H6A	109.3	C11—C14—H14C	109.5
C5—C6—H6B	109.3	H14A—C14—H14C	109.5
C7—C6—H6B	109.3	H14B—C14—H14C	109.5
H6A—C6—H6B	108.0		
			
C2—N1—C1—C4	−2.7 (3)	N2—C3—C4—C5	178.08 (16)
C2—N1—C1—S1	179.11 (13)	C1—C4—C5—C6	−99.1 (2)
C8—S1—C1—C4	162.19 (15)	C3—C4—C5—C6	80.5 (2)
C8—S1—C1—N1	−19.57 (16)	C4—C5—C6—C7	174.6 (2)
C3—N2—C2—O1	175.69 (18)	C1—S1—C8—C13	123.21 (15)
C3—N2—C2—N1	−3.8 (3)	C1—S1—C8—C9	−62.23 (17)
C1—N1—C2—O1	−176.24 (17)	C13—C8—C9—C10	−2.0 (3)
C1—N1—C2—N2	3.3 (2)	S1—C8—C9—C10	−176.42 (15)
C2—N2—C3—O2	−176.34 (17)	C8—C9—C10—C11	1.8 (3)
C2—N2—C3—C4	3.4 (3)	C9—C10—C11—C12	0.3 (3)
N1—C1—C4—C3	2.1 (3)	C9—C10—C11—C14	−178.5 (2)
S1—C1—C4—C3	−179.72 (13)	C10—C11—C12—C13	−2.2 (3)
N1—C1—C4—C5	−178.32 (17)	C14—C11—C12—C13	176.6 (2)
S1—C1—C4—C5	−0.1 (2)	C9—C8—C13—C12	0.1 (3)
O2—C3—C4—C1	177.39 (17)	S1—C8—C13—C12	174.76 (16)
N2—C3—C4—C1	−2.3 (2)	C11—C12—C13—C8	2.0 (3)
O2—C3—C4—C5	−2.2 (3)		

### Hydrogen-bond geometry (Å, º)

**Table:**

*D*—H···*A*	*D*—H	H···*A*	*D*···*A*	*D*—H···*A*
N2—H2*N*2···O1^i^	0.84 (2)	1.98 (2)	2.815 (2)	173 (2)
N1—H1*N*1···O2^ii^	0.79 (2)	2.17 (2)	2.8988 (18)	155 (2)

Symmetry codes: (i) −*x*+1, −*y*, −*z*+1; (ii) *x*, −*y*+1/2, *z*+1/2.
